# Book Reviews and Notices

**Published:** 1888-08

**Authors:** 


### BOOK REVIEWS AND NOTICES.

ANNUAL OF THE UNIVERSAL MEDICAL SCIENCES: A Yearly Report of the Progress of the General Sanitary Sciences Throughout the World. Edited by CHARLES E. SAJAUS, M. D., Lecturer on Laryngology and Rhinology in Jefferson Medical College, Philadelphia, etc., and seventy associate editors, assisted by over 200 corresponding editors, collaborators, and correspondents. Illustrated with chromo-lithographs, engravings, and maps. 1888. Philadelphia and London: F. A. Davis, publisher.

The object of this work, as stated in the preface, "is to collate the progressive features of medical literature at large, and clinical data from countries in which no literature exists, and to present the whole once a year in a continued form prepared by writers of known ability. As such, it is expected to become a helpmate to the practitioner in his efforts to relieve suffering and to assist the investigator by corelating facts."

The work for this year appears in five octavo volumes of about 350 pages each. The table of contents of each volume is upon the back of the cover, for the convenience of ready reference. At the end of the work there are three indices in parallel columns, viz., a general index, a therapeutic index, and a bibliographical index.

The articles give abundant evidence that the work has been carefully done.

The editor is to be congratulated that in so large an undertaking he has been able to so nearly accomplish the design. His efforts will be gratefully appreciated by a host of our busy confrères who have not the time to sift the wheat from the chaff. The editor recognizes the fact that a publication of this nature appearing only once in a year greatly detracts from its own usefulness by often not presenting material till long after it has become public property. To overcome this drawback a quarterly journal is published in connection with the annual —*The Satellite*—which is intended to present to the reader all the new important facts published during the preceding three months, and especially those bearing upon the prevalent diseases of the season.

The annual and its satellite have fairly started upon their yearly and quarterly revolutions. We hope that each succeeding round will bring them to us more symmetrical, more nicely balanced, more nearly perfect.

A SYSTEM OF OBSTETRICS BY AMERICAN Authors. Edited by BARTON COOKE HIRST, M. D. Vol. I. Philadelphia: Lea Brothers & Co. 1888.

The first volume of this publication, including the contributions by Drs. Engelmann, Hirst, Jaggard, Busey, Penrose, Reeves, and Parvin, has been issued.

The history of obstetrics could not have been intrusted to abler hands. Dr. Engelmann has produced an admirable summary of his subject. The physiology and histology of ovulation, menstruation, and fertilization, the development of the embryo, have been fully and ably treated of by Dr. H. Newell Martin. Dr. Hirst has complete chapters on the development, anomalies, monstrosities, diseases, and premature expulsion of the ovum. Dr. Jaggard writes with his usual clearness of the physiology, pathology, signs, and differential diagnosis of pregnancy. It is a disappointment that so learned an obstetrician as Dr. Jaggard could express himself no more positively in reference to the question of changes in the neck of the gravid uterus than to say "The weight of evidence seems to us very much in favor of Bandl's modified theory." It is now seventy-five years since Madame Boivin wrote the sentences which follow:

"In fact, the neck of the uterus at term alone forms more than a third of the cavity of the uterus."

"Accoucheurs are continually speaking of the internal orifice in the last months of pregnancy. They forget that the os internum has long been effaced."

"The circumference of the uterus at term, measured at the top of the uterine portion of the neck (which is then five inches above the external orifice), is about thirteen inches."

It is fifty years since Guillemot, in referring to hour-glass contraction, wrote:

"When the hand is introduced the cervix is found projecting into the vagina, and so disfigured that it resembles a section of large intestine; but about five or six inches above this the finger is arrested by a kind of stricture which is the wrinkled and contracted internal orifice."* The views of changes occurring in the cervix deducible from these two authors are perfectly consistent, it seems to us, with every fact regarding the anatomy, physiology, and pathology of the uterine neck, before, during, and after labor. And yet, such is the force of preconceived opinion that to-day not a single author of a work on obstetrics has completely emancipated himself from such apparently irrational and inconsistent theories as were put forth by Stoltz in 1826.

The views of Boivin and Guillemot, current in Chicago for the last fifteen years, have been accepted with all their conclusions by a number of physicians of this city. So far as is known, nothing has been observed in their practice to contravene their adopted opinions. It is to be hoped that in the next volume of the American System of Obstetrics, the writer upon placenta prævia, taking a hint given by Madame Lachapelle three-quarters of a century ago, will locate the placenta below the os internum.

The conduct of labor and the management of the puerperal state are treated of in an able and interesting manner by Dr. Samuel C. Busey, in a style that pleasantly recalls the writings of Dr. Meigs.

The mechanism of labor, and the treatment of labor based on the mechanism, are treated of by Dr. R. A. F. Penrose. Though these subjects are presented in a manner vigorous, thoughtful, and fresh, many readers will doubtless have occasion to dissent from some of the positions taken by the author. For example, it would seem to us that Dr. Penrose takes an exaggerated view of the difficulties and dangers of occipito-posterior positions. We object to the advice given by the author in reference to the conduct of the obstetrician where he has not succeeded in delivering in these cases by the several methods suggested After a consultation, a mere formality, he is advised to perforate at once, or "to abandon, instantly, the case." This conduct might be very proper for Dr. Penrose, but for the average practitioner it would be impolitic, unwise, and censurable. So far, nothing has been demonstrated in the suppositive case except that the means adopted have not succeeded in the hands of the attendant in charge. To the consultant, as skillful as Dr. Penrose, success might follow any one of the means with which the inexperienced operator has failed. In midwifery there is no call for dogmatism of opinion and obstinacy of purpose. Nor, it may be added, is there in the exigencies of the lying-in chamber need of "heroism" or "heroics"; better than these is calm courage.

The article on the use of anæsthetics in labor, by Dr. J. C. Reeve, is excellent. Dr. Parvin writes the chapters on the anomalies of the forces of labor with his usual ability.

This volume is replete with facts of the greatest interest to the obstetrician, and we commend it accordingly.

* Cazeaux.

PRACTICAL ELECTRO-THERAPEUTICS. By WILLIAM F. HUTCHINSON, M. D. 8vo. Pp. 247 Philadelphia: Records, McMullin & Co. 1888.

The author's motto, "That he who runs may read," is well exemplified in this book. It is a concise treatise upon electro-therapeutics stripped of theoretical considerations. The relative merits and demerits of the different varieties of batteries and electrical apparatus, and the manner of using the various appliances and the results to be expected from their use, are briefly considered. The chapters upon the special medical and surgical uses of electricity contain many clinical notes. The author has availed himself of his rich experience to show how and under what circumstances treatment should be conducted, often entering into the minutest details in explaining the technique.

The book will be a very useful one to the many members of our profession who have not been able to spare the time to master this subject, and it will certainly be read with pleasure and profit by the many who are already familiar with the facts of which it treats.

HENRY'S POSOLOGICAL AND THERAPEUTIC Tables, containing the Doses, Action, and Uses of the Medicines in the British Pharmacopœia, with poisons. Third edition. Revised and enlarged. Pp. 83. 12mo. Edinburgh: Maclachlan & Stewart. London: Simpkin, Marshall & Co. 1888.

Is a small dose-book based upon the British Pharmacopœia It has in addition to the list of preparations of drugs with doses, action, and uses, a catalogue list of diseases with drugs that have been found useful in each, and a list of poisons, their action and their antidotes.

